# A Quasi-Distributed Crack Sensor Based on Weakly Coupled Vertical U-Shaped Ring Array

**DOI:** 10.3390/s25092852

**Published:** 2025-04-30

**Authors:** Chenjie Chu, Jiayi Huang, Xuan Xie, Jun Zhang

**Affiliations:** School of Information Engineering, Guangdong University of Technology, Guangzhou 510006, China; 2112203018@mail2.gdut.edu.cn (C.C.); 2112403094@mail2.gdut.edu.cn (J.H.); 2112303084@mail2.gdut.edu.cn (X.X.)

**Keywords:** quasi-distributed sensor, transmission line, vertical U-shaped ring, crack detection, structural health monitoring

## Abstract

Cracks are common defects in metallic components, the presence of which can significantly affect service life and operational stability. Sensors based on electromagnetic resonators have relatively high sensitivity; however, they are limited in size, which restricts their coverage and makes large-area monitoring unattainable. The uneven internal field distribution within the resonator is a critical factor contributing to sensitivity variation at different locations. In this study, a vertical U-shaped ring structure is excited using a microstrip line. This allows the sensor to achieve large-area monitoring while maintaining sensitivity. The shift in resonance frequency is investigated and extracted as a characteristic feature for crack identification. The sensitivity of the measurement is 0.95 GHz/mm^2^ for depth and 0.685 GHz/mm^2^ for width. The proposed sensor can be used to detect potential cracks in metal structures.

## 1. Introduction

Intelligent aircraft structure is a revolutionary technology that integrates sensors into the aircraft structure, enabling the aircraft not only to withstand external loads but also to possess self-sensing and self-diagnostic capabilities [1]. During service, precise structural deformation monitoring and control play a crucial role in ensuring flight efficiency and safety. The development and propagation of cracks have adverse effects on the structural integrity and performance of metal components. Therefore, the detection of micro-cracks throughout the entire life cycle of metal structures is crucial for preventing catastrophic failures in engineering components and structures [2]. To guarantee structural safety, structural health monitoring (SHM) technologies have been extensively researched in recent decades [3], which can be used to collect real-time continuous or intermittent data, allowing for the prediction of the structure’s health status and future performance, thereby optimizing maintenance [4,5].

SHM technology can be categorized into several types, including optical fibers [6,7], ultrasound [8], vibration [9,10], piezoelectric impedance [11,12], and microwave methods [13,14]. Microwave methods can effectively penetrate the metallic surface through coating, demonstrating high sensitivity. Additionally, they can be integrated with other systems, making them suitable for defect detection in various applications. For complex structures, comprehensive monitoring requires the collaborative operation of multiple sensors. However, ensuring accuracy while optimizing sensor placement remains a significant challenge in the development of SHM systems.

For individual microwave sensors, research has focused on the optimization of frequency response and loss characteristics to achieve high sensitivity detection through material improvement and structural design. The research on sensor arrays has just started [15], and is expected to realize high sensitivity detection over a wide range by designing the size and distribution of the units. Precise control of the size of resonant units through sensor arrays enables the realization of a crack location [16]. However, there are still challenges in sensitivity and consistency due to the inhomogeneous field distribution brought about by multi-unit coupling.

The bound state of the continuum (BIC) enables the design of optical resonant structures with a high-quality factor using low-loss structures that are tuned from local to non-local through a change in resonance modes, resulting in the transition from quasi-isolated localized resonances to extended resonance modes; this involves strong interactions between neighboring structural units to achieve a high-Q resonance in the plasma meta-surface [17,18]. By controlling the height of the holder (linker), the transition of the light and dark modes of the localized plasma resonance (LSPR) between the localized and non-localized states is induced, thus enhancing the spatial coherence while increasing the sensitivity.

The resonator size limits the coverage of highly sensitive detection, with a small hot spot region and low spatial resolution for sensor crack detection. The high sensitivity detection and local field confinement capabilities of electromagnetic sensors are highly correlated. The aim of this paper is to study the high sensitivity sensors with an extended detection range, so the research breakthrough is carried out from the perspective of local field confinement and spatial uniformity. Based on the above analysis, this paper proposes a transmission line structure based on an interleaved loaded vertical U-shaped ring. In contrast, by reducing the height of the pillars, the mutual coupling between units is suppressed, thereby improving the detection sensitivity. The design of interleaved distribution can realize a spatially homogeneous field distribution, which can be used to improve spatial resolution in crack detection, forming the so-called quasi-distributed sensor. The paper is organized as follows. Section 2 analyzes the design and operating principle of the sensor. Section 3 investigates the testing and results analysis. Section 4 summarizes the contribution of this work.

## 2. Sensor Design

This section describes the cell design and array structure. First, an interleaved vertical U-shaped ring (VUR) loading transmission line structure is proposed. Then, the design considerations of the interweaving distribution are investigated from the perspective of the spatial resolution of the crack. A relatively uniform field distribution along the wave propagation direction is achieved by appropriate interleaving spacing, which attenuates the effect of the relative position of the cracks on the detection consistency. Then, the sensing mechanism is analyzed from qualitative and quantitative perspectives. Finally, the crack detection performance is evaluated.

### 2.1. Sensor Structure

**Figure 1:**
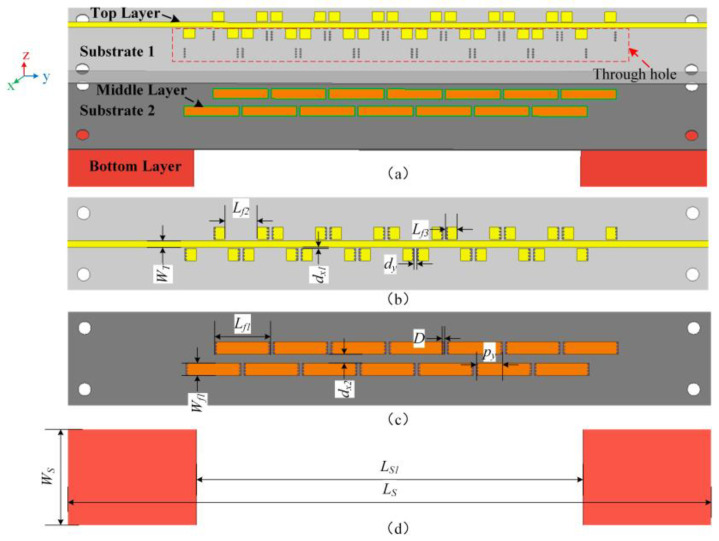
Schematic structure of the sensor (**a**) 3D structure of the sensor, (**b**) the top layer, (**c**) the middle layer and (**d**) the bottom layer.

**Figure 2:**
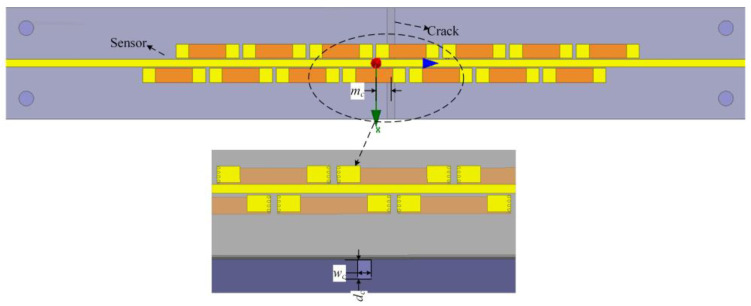
The simulation setup for the crack detection using the proposed sensor, where *w_c_* represents the width of the crack, *d_c_* represents the depth of the crack, and *m_c_* represents the position of the crack.

The structure of the proposed sensor is shown in Figure 1 and the information about the crack size and the relative position between the sensor and the crack are shown in Figure 2. In the design, each VUR consists of three parts, namely, an open strip on the upper layer, a complete strip on the lower layer, and metal through-holes connecting the strips on the upper and lower layer. The upper strip is in the same layer as the microstrip line and the lower strip is located in the middle layer between the ground and the microstrip line. The VUR is distributed on both sides of the microstrip line in an interwoven arrangement to be easily excited by the edge magnetic field of the microstrip line.

The proposed sensor has a total of two layers of dielectric substrates: substrate 1 and 2, the material of which are F4BM300, with a dielectric constant of 3, a loss tangent of 0.0017, and a thickness of 0.2 mm. The total dimensions of the proposed sensor are 100 mm × 15 mm × 0.4 mm. The dimensions of the VUR are only 8.5 mm × 1.9 mm × 0.2 mm. The detailed dimensions are shown in Table 1.

The microstrip line is a broadband structure that provides uniform excitation to the VURs on both sides, while the perturbations to the VURs can be coupled to the microstrip line and thus be measured through the transmission coefficient. In addition, other unaffected resonator units can be used as a reference for defect characterization.

### 2.2. Sensing Mechanism

The generation of cracks perturbs the electric/magnetic field distribution of the resonator. Perturbation theory [19] describes the sensing mechanism of electromagnetic sensors, and the reason for the frequency point shift caused by crack changes is the change in the magnetic–electrical energy difference of the resonator brought about by the appearance of cracks. The changes between the resonant frequency shift and the energy storage can be obtained from the equation:

(1) $$\frac{w-w_{0}}{w}=\frac{\Delta W_{m}-\Delta W_{e}}{W_{m}+W_{e}}$$

Among them, $\Delta W_{m}$ represents the difference in the stored magnetic energy between the original state and the perturbed state, $\Delta W_{e}$ represents the difference in the stored electric energy between the original state and the perturbed state, $W_{m}$ represents the magnetic energy stored in the original state of the resonator, and $W_{e}$ represents the electric energy stored in the original state of the resonator.

In other words, cracks of different sizes cause different magnetic–electric energy differences, which correspond to different resonant frequency points. Since the electric/magnetic field inside an electromagnetic resonator is sinusoidal, the magnetic–electric energy difference due to a crack of the same size varies at different locations, so the detection sensitivity is affected by the relative location of the crack, resulting in a decrease in the reliability of the detection results. Therefore, the crack detection performance of a single resonator is limited.

**Figure 3:**
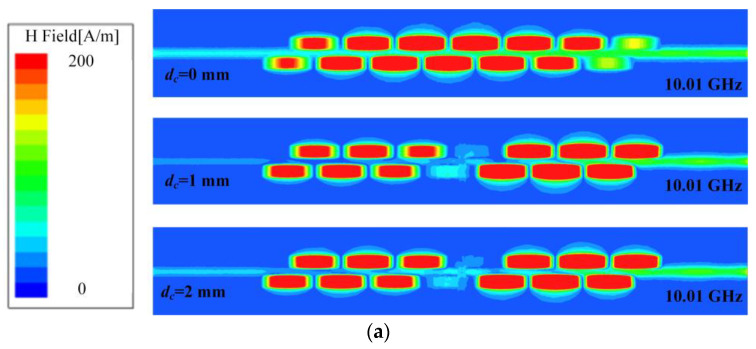

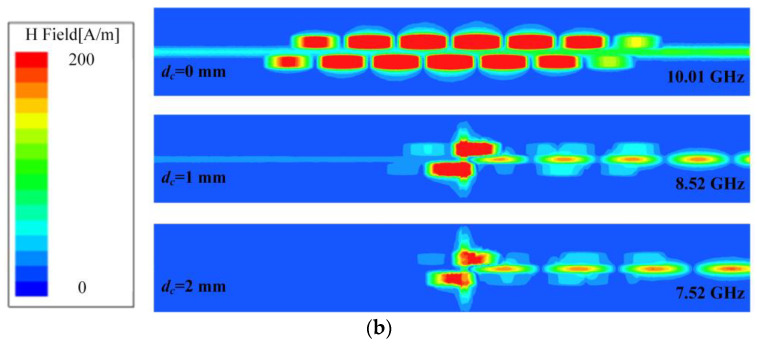
Magnetic field distributions in the healthy state and at the presence of cracks. (**a**) at 10.01 GHz and (**b**) at resonance frequencies.

Electromagnetic waves propagate along the microstrip line and reflect at the crack due to the discontinuity of the boundary conditions, and the forward and backward waves will meet in the formation of standing waves. Figure 3 shows the magnetic field distribution along the traveling wave direction. When the crack appears, the magnetic field at the VUR, which is directly affected by the crack, is obviously weakened, which is caused by the resonance point being shifted to a lower frequency. It can be seen that the VUR structure has a relatively smooth field distribution in a single resonator, owing to the other sinusoidal parts being folded into the strip on the upper layer.

**Figure 4:**
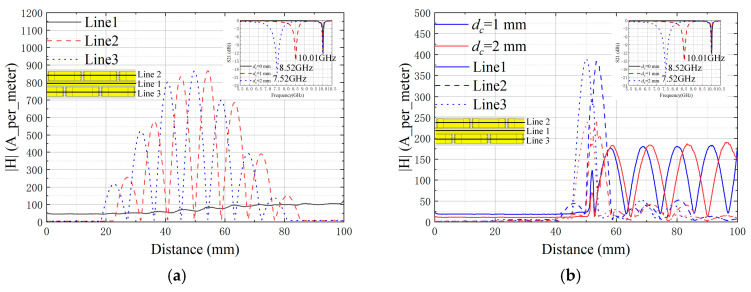
Comparison of sensor magnetic field amplitude, where the line style differentiation denotes varying extraction positions. (**a**) Healthy state (10.01 GHz) and (**b**) at the presence of cracks (8.52 GHz–7.52 GHz), where the color variations of the lines correspond to distinct crack depths.

Figure 4 shows a comparison of the magnetic field amplitude in the healthy state and at the presence of cracks by extracting the magnetic field (on the surface of the ground plane) from the three lines. As can be observed from Figure 3a, the VUR can be excited through coupling, and the magnetic field after resonance is confined and enhanced within the unit area. The presence of cracks affects the distribution of the magnetic field. As shown in Figure 3b, the amplitude of the magnetic field under the VUR drops sharply after the growth of cracks.

### 2.3. Sensing Performance

To reveal the sensing properties of the interwoven cells, the same size of crack is used to pass through the upper cell, the lower cell, and the interwoven region respectively. Accordingly, ’up’, ’down’, and ’total’ are used to denote the different ways of crack crossing.

**Figure 5:**
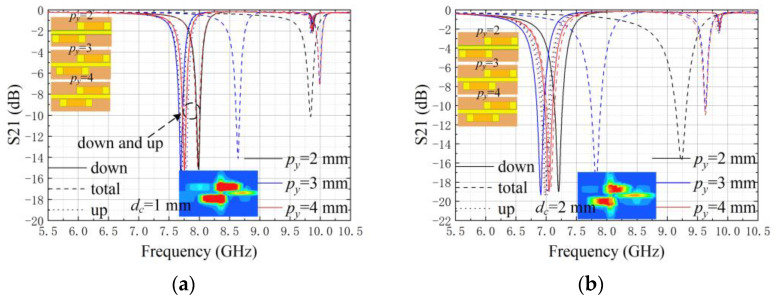
Transmission coefficients versus frequency for a single interwoven structure at a fixed width (*w_c_* = 1 mm). (**a**) *d_c_* = 1 mm and (**b**) *d_c_* = 2 mm.

Figure 5 represents the simulated transmission coefficients when the interleaving distance (*p_y_*) of the upper and lower units varies from 2 to 4 mm. It can be proven that a proper interleaving distance can weaken the influence of the crack location on the detection consistency, where a *p_y_* of 4 mm can be selected in the following study.

**Figure 6:**
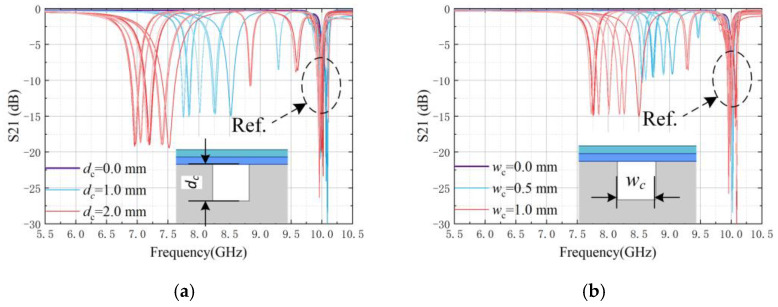
Transmission coefficients under variations of crack position (*m_c_*) from 0–10 mm in 1 mm steps (different gradients of the same color). (**a**) For a fixed crack width (*w_c_*) of 1 mm, the crack depth (*d_c_*) varies from 0.0 mm–2.0 mm in the step of 1 mm and (**b**) for a fixed crack depth (*d_c_*) of 1 mm, the crack depth (*w_c_*) varies from 0.0 mm–1.0 mm in the step of 0.5 mm.

The simulated transmission coefficients of the proposed sensor are shown in Figure 6. In the healthy state, the VUR resonates at 10.01 GHz. When a crack is generated, the resonance frequency of the affected unit will be shifted to the lower frequency region.

**Figure 7:**
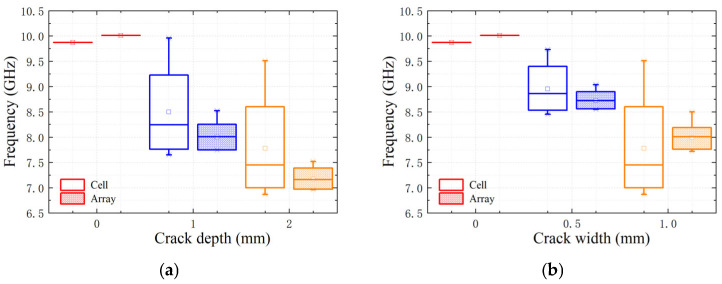
Box line plots of the simulated resonance frequency point as a function of crack position (*m_c_*). (**a**) Variation of resonant frequency points with depth change for a fixed width of 1 mm and (**b**) Variation of resonant frequency points with width change for a fixed depth of 1 mm.

The box-and-line plot of the resonant frequencies extracted from the transmission coefficients is shown in Figure 7. The average sensitivity of the depth characterization for a 1 mm crack width is 1.42 GHz/mm^2^; the average sensitivity of the width characterization for a 1 mm crack depth is 1.01 GHz/mm^2^. It can be seen from the frequency distribution of the line plots that, for the same width (depth), the resonant frequency points that vary with the depth (width) are monotonically shifted towards lower frequencies, which is due to the dominance of magnetic perturbation. The precision of the sensor with unknown information in the crack location is better than the single resonator unit.

## 3. Measurements and Results

### 3.1. Test Setup

**Figure 8:**
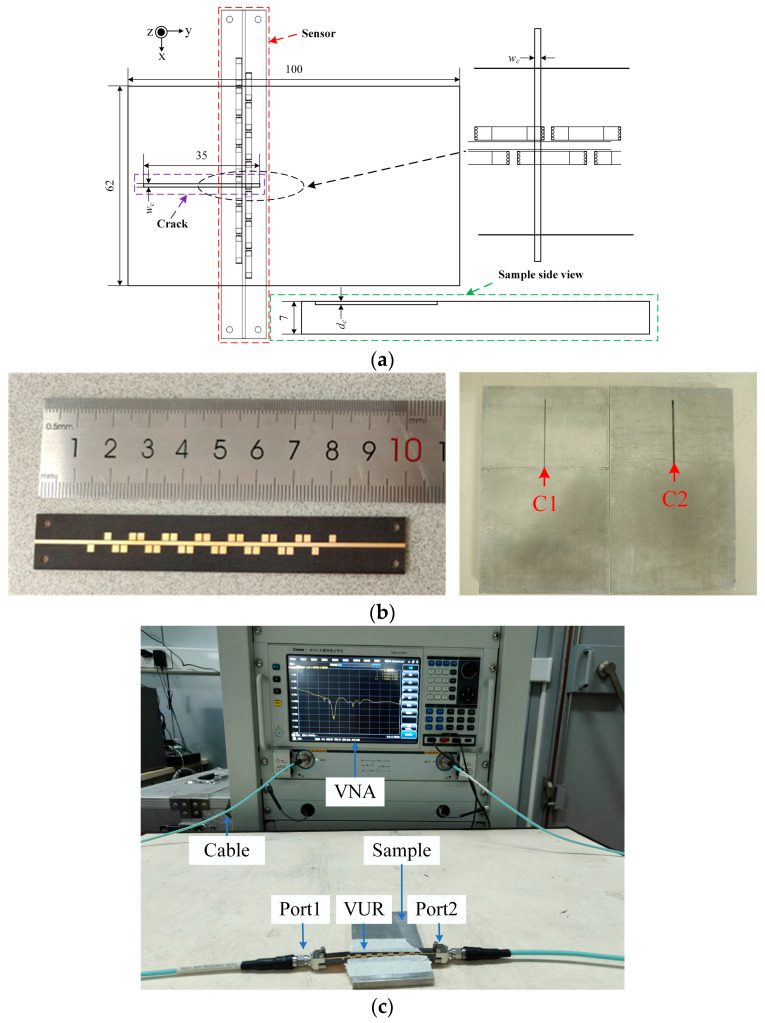
Test setup (**a**) Measurement schematic, (**b**) Photographs of prototype sensors and sample, and (**c**) Test setup. Unit: millimeter.

The tests were performed at room temperature and under dry conditions. The schematic diagram of the proposed sensor and the test setup are shown in Figure 8. Two aluminum alloy samples with dimensions of 100 mm × 62 mm × 7 mm were fabricated using the Computer Numerical Control (CNC) method and their dimensions are detailed in Figure 8a, with a crack width of 1 (0.5) mm. The material of the sensors is F4BM300, the thickness of the substrate is 0.2 mm in both cases, and the thickness of the copper is 0.035 mm. The sensor is prototyped and shown in Figure 8b. Measurements were performed using a Ceyear3672C’s two-port vector network analyzer (VNA), with the sensor mounted directly on the surface of the sample under test.

### 3.2. Results and Discussion

**Figure 9:**
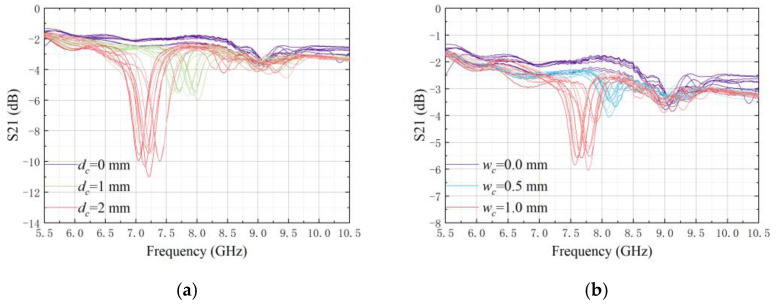
Measured transmission coefficients under variations of crack position (*m_c_*) from 0–10 mm in 1 mm steps (different gradients of the same color). (**a**) Crack depth (*d_c_*) varies from 0.0 mm–2.0 mm in the step of 1 mm at a fixed crack width (*w_c_*) of 1 mm, and (**b**) crack depth (*w_c_*) varies from 0.0 mm–1.0 mm in the step of 0.5 mm at a fixed crack depth (*d_c_*) of 1 mm.

The measured transmission coefficients of the proposed sensor are shown in Figure 9. The measurement results are in general agreement with the simulation ones. Compared with the simulation results, the measurement results are centrally shifted to lower frequencies by about 1 GHz. These errors mainly originate from the processing and manufacturing errors of the PCB and the small air gaps between the sensor and the sample to be tested.

**Figure 10:**
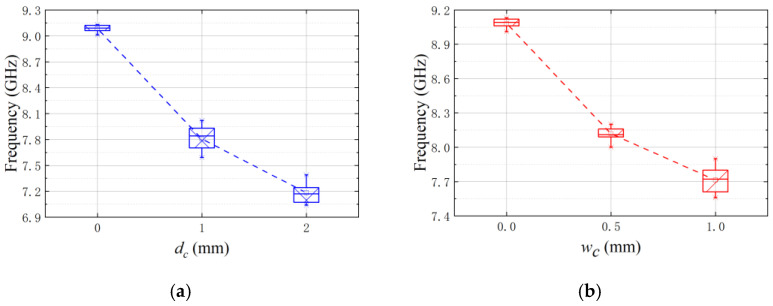
Boxplots of resonant frequency points. (**a**) Variation of resonant frequency points with depth change at a fixed width of 1 mm and (**b**) Variation of resonant frequency points with width change at a fixed depth of 1 mm.

The box plots of the resonant frequencies are shown in Figure 10. The average sensitivity of the measured depth variation for a 1-mm-width crack is 0.95 GHz/mm^2^; the average sensitivity of the measured width variation for a 1-mm-depth crack is 0.685 GHz/mm^2^. The resolution of the sensor can be 1 mm × 0.5 mm. The precision is 21.7%/F.S. The spatial resolution of the sensor can be improved by the interweaving method.

The performance of the proposed sensor is compared with other works in Table 2. The sensitivity is defined as S = ∆f/∆c, and the relative sensitivity is defined as RS = S/f_0_, with f_0_ being the operating frequency of the resonator without cracks. The sensor provides high sensitivity and flexibility compared to the ORWG method. The sensor has better detection consistency, i.e., insensitivity to crack location, and is capable of achieving long coverage (or high spatial resolution) compared with CSRR or other meta-material inspired resonator units. Therefore, the proposed sensor can maintain competitive performance.

## 4. Conclusions

There is a risk of missing detection due to the non-uniform field distribution within the electromagnetic sensors owing to the boundary condition, which limits the application scenario and complexity in the installation and placement. From the perspective of the electromagnetic wave, a relatively uniform field distribution along the propagation direction is achieved by loading interlaced VUR cell units. The vertical configuration suppresses the influence of mutual coupling between units, thus improving the detection sensitivity. Therefore, the detection sensitivity of the crack depth (width) is 0.95 (0.685) GHz/mm^2^ and the minimum detectable crack size is 0.5 mm × 1 mm.

This paper offers a design paradigm for improving spatial resolution by turning an electromagnetic resonator from point-based to quasi-distribution. The short-distance coverage of a single electromagnetic resonator is extended by arraying the technique via an interweaving form; the spatial resolution for the crack detection is comparably verified. In subsequent research, the non-local resonance effect can be used to further improve spatial resolution.

## Figures and Tables

**Table 1:** Detailed dimensions of the sensor. Unit: millimeter.

*W_S_*	*L_S_*	*L_S1_*	*L_f1_*	*L_f2_*
15	100	60	8.5	5
*W_f1_*	*d_y_*	*d_x1_*	*d_x2_*	*W_T_*
1.9	0.5	0.2	1.4	1
*p_y_*	*L_f3_*	*D*		
interweaving distance	1.75	0.25		

**Table 2:** Comparison of the performance of this work with other sensors.

Ref.	Structure (Feed + Resonator)	Frequency f_0_ (GHz)	Sensitivity (GHz/mm^2^)	RS	Resolution	Long Coverage
[20]	ORWG + Dielectric resonator	16.8	depth: 0.71 width: 1.24	0.042 0.074	1 mm × 1 mm	N
[21]	CSRR + TL	5.05	1.25	0.247	0.2 mm × 1 mm	N
[22]	Chipless + Patch	4.7	0.1343	0.029	0.1 mm × 1 mm	N
This work	VUR + TL	9.08	depth: 0.95 width: 0.685	0.104 0.075	1 mm × 0.5 mm	Y

## Data Availability

Data are contained within the article.

## Abbreviations

The following abbreviations are used in this manuscript:

SHM	Structural health monitoring
VUR	Vertical U-shaped ring
BIC	Bound states in the continuum
ORWG	Open rectangular waveguide
